# Associations between dietary composition, water intake, urine pH, and cardiometabolic indicators among young Saudi women

**DOI:** 10.1371/journal.pone.0355378

**Published:** 2026-08-13

**Authors:** Jozaa Z. ALTamimi, Dalal H. Aljabryn, Ghadah Saud Alnooh, Jwaher Haji Alhaji, Eman Alamri, Abdelaziz Hendy, Nora A. AlFaris

**Affiliations:** 1 Department of Sports Health, College of Sports Sciences & Physical Activity, Princess Nourah bint Abdulrahman University, Riyadh, Saudi Arabia; 2 Department of Health Sciences, College of Health and Rehabilitation Sciences, Princess Nourah bint Abdulrahman University, Riyadh, Saudi Arabia; 3 Department of Health Sciences, College of Applied Studies, King Saud University, Riyadh, Saudi Arabia; 4 Department of Food Science and Nutrition, University of Tabuk, Tabuk, Saudi Arabia; 5 Department of Maternal and Child Health Nursing, College of Nursing, Qassim University, Buraydah, Saudi Arabia; 6 Pediatric Nursing, Faculty of Nursing, Ain Shams University, Cairo, Egypt; 7 Nursing Services Department, Qassim University Medical City, Buraydah, Saudi Arabia; University of Rwanda College of Medicine and Health Sciences, RWANDA

## Abstract

**Background:**

Cardiometabolic disorders continue to rise globally, yet limited evidence exists regarding the associations between dietary composition, hydration status, urine pH, and cardiometabolic risk indicators in young Saudi women.

**Aim:**

To investigate the associations between dietary composition, water intake, urine pH, and cardiometabolic indicators among young Saudi women.

**Methods:**

A cross-sectional analytical study was conducted among 135 Saudi women aged 19–30 years in Riyadh, Saudi Arabia. Data were collected remotely using a structured, self-administered electronic questionnaire under home-based conditions. Anthropometric measurements, blood pressure, water intake, dietary intake, and urine pH were assessed following standardized instructions provided to participants. Dietary intake was evaluated using repeated 24-hour dietary recalls and analyzed using Food Processor software (ESHA Research, Version 7). Statistical analyses included descriptive statistics, Pearson correlation analysis, and multiple linear regression models to examine associations between dietary intake, hydration status, and cardiometabolic risk indicators.

**Results:**

Central adiposity was present in 26.6–35.5% depending on the index. Elevated diastolic blood pressure was observed in 72.6% of participants. Only 8.9% consumed ≥8 cups of water/day. Higher energy, sodium, and omega-6 intake were positively associated with systolic blood pressure, while carbohydrate and phosphorus intake showed inverse associations. Energy intake was positively associated with diastolic blood pressure, and urine pH showed weak positive correlations with soluble fiber and thiamin.

**Conclusion:**

Dietary composition and hydration status were significantly associated with blood pressure and urine pH among young Saudi women. Higher energy and sodium intake were associated with higher blood pressure levels, while carbohydrate intake showed inverse associations with blood pressure outcomes. In addition, dietary fiber, selected micronutrients, and unsaturated fatty acids demonstrated variable associations with cardiometabolic risk indicators. Regarding urine pH, energy intake was inversely associated, whereas carbohydrate, omega-6, and magnesium intake showed significant associations. These findings highlight important diet–health relationships; however, they should be interpreted as exploratory associations rather than causal effects due to the cross-sectional design of the study.

## Introduction

Rapid socioeconomic and lifestyle transitions have substantially altered dietary behaviors worldwide, contributing to an increasing burden of non-communicable diseases (NCDs), which account for approximately 71% of global deaths. Among these conditions, cardiovascular diseases, including hypertension, remain the leading contributors to morbidity and mortality [1,2].

Metabolic syndrome and related cardiometabolic risk factors have been widely reported across diverse populations and ethnic groups in epidemiological studies and reports from international health organizations [3]. In Gulf countries, an increasing prevalence of cardiometabolic risk factors has been documented, including obesity, central adiposity, and hypertension, with reported rates of approximately 17% in Oman, over 40% in the United Arab Emirates, and more than 39% in Saudi Arabia [4]. Among these risk factors, hypertension represents a major public health concern due to its strong association with cardiovascular morbidity and mortality, including heart failure and stroke [5]. Even modest reductions in population-level blood pressure have been shown to significantly reduce cardiovascular risk [6].

Diet is a fundamental determinant of cardiometabolic health, influencing both the development and prevention of NCDs [1]. Strong evidence indicates a positive association between dietary sodium intake and blood pressure [6], while excessive consumption of energy-dense, nutrient-poor foods contributes to the increasing prevalence of obesity and related metabolic disturbances [2]. In addition, hydration status is an important indicator of health, as adequate water intake is essential for maintaining renal function, fluid balance, and metabolic homeostasis [7]. Furthermore, urine pH has been identified as a potential marker influenced by overall dietary composition, including macronutrient and micronutrient intake [8].

Despite extensive global research on diet and cardiometabolic health, evidence specifically focusing on young Saudi women remains limited. In particular, there is a gap in studies integrating dietary intake, hydration status, anthropometric measures, blood pressure, and urine pH within a single analytical framework in this population. Therefore, the present study aimed to examine the associations between anthropometric characteristics, hydration status, dietary composition, blood pressure, and urine pH among young Saudi women. The findings may provide exploratory evidence to inform future nutritional interventions and support hypothesis generation for longitudinal research within the context of national public health priorities, including Saudi Vision 2030.

## Methods

### Study design

This study employed a cross-sectional analytical design to investigate the associations between dietary composition, water intake, urine pH, and cardiometabolic indicators among young Saudi women. The cross-sectional approach was selected to assess relationships between variables at a single point in time and to identify potential dietary predictors of blood pressure and urine pH. This design is appropriate for exploring population-level associations and generating hypotheses for future longitudinal research.

### Subjects

The study included a total of 135 young Saudi women aged between 19 and 30 years. Participants were recruited using a convenience sampling technique through electronic invitations distributed via social media platforms.

Inclusion criteria: Female gender, Age between 19 and 30 years, Residence in Saudi Arabia, Willingness to participate and provide informed consent. Exclusion criteria: Pregnancy or lactation, History of chronic diseases (e.g., diabetes, hypertension, kidney disease), Current use of medications affecting blood pressure or metabolism

### Sample size

The sample size of 135 participants was determined based on feasibility and accessibility of the target population of young Saudi women within the study setting. The study followed a pragmatic sampling approach commonly used in cross-sectional nutritional epidemiology studies where multiple dietary exposures are assessed simultaneously. To ensure adequacy for multivariable analysis, the sample size was evaluated against established recommendations for multiple linear regression, which suggest a minimum ratio of 10–15 participants per predictor variable. Given that the regression models included between 7 and 9 predictors, the achieved sample size exceeded these recommendations.

In addition, a post-hoc assessment of statistical power was conducted based on the observed effect sizes from the regression models (R^2^ ranging from 0.275 to 0.40), indicating that the study had adequate statistical power (>0.80) to detect medium-to-large effects. This supports the suitability of the sample size for the analytical objectives of the study.

### Setting

The study was conducted in Riyadh, Saudi Arabia, using a remote web-based data collection design. Data were collected through a structured, self-administered electronic questionnaire, allowing participation from young women residing within the city. Anthropometric measurements, blood pressure readings, dietary intake data, and urine pH values were obtained under home-based conditions. Participants followed standardized, step-by-step instructions provided by the research team to ensure consistency in data collection procedures.

Urine pH was assessed using provided test strips, and participants were instructed to collect a midstream morning urine sample and record the results in the questionnaire. This remote data collection approach facilitated feasible and standardized data acquisition while ensuring accessibility within the target urban population.

### Tools

#### Anthropometric measurements and cardiometabolic health.

Participants self-measured weight, height at home following standardized written instructions. Body mass index (BMI) was calculated as weight (kg) divided by height squared (m^2^) and classified according to WHO criteria: underweight (<18.5 kg/m^2^), normal (18.5–24.9 kg/m^2^), overweight (25.0–29.9 kg/m^2^), and obese (≥30.0 kg/m^2^). Waist circumference (WC) and hip circumference (HC) were measured using a flexible tape, with WC measured midway between the lower rib margin and iliac crest, and HC at the widest part of the buttocks. Waist-to-hip ratio (WHR) and waist-to-height ratio (WHtR) were subsequently calculated as indicators of central obesity. Participants measured blood pressure 3–4 times using a home sphygmomanometer, and the average reading was used for analysis. For additional details on the questionnaires and laboratory procedures used, refer to the NHANES website [9,10].

#### Demographic and socioeconomic data.

Participants self-reported their demographic and lifestyle characteristics. Daily water intake was self-reported by participants and categorized into three groups: 2–5 cups, 5–7 cups, and 8 or more cups per day (Table 1).

**Table 1:** Characteristics of the study sample (n = 135).

		Frequency	%
BMI	< 18	45	33.30
	18.5–24.9	35	25.90
	24.9–29.9	32	23.70
	≥30	23	17.03
WHR	<0.85	99	73.40
	>0.85	36	26.60
WHtR	<0.4	22	16.29
	0.40–0.49	65	48.14
	0.50–0.59	40	29.62
	≥0.6	8	5.92
DBP mmHg	<80	37	27.40
	80–89	80	59.30
	≥90	18	13.30
SBP mmHg	<120	77	57.03
	120–129	42	31.11
	130–139	8	5.92
	≥140	8	5.92
Sodium Intake	less than 2000 mg/day	94	69.62
	2000 or more	41	30.37
Water Intake per Day (cups)	2–5	60	44.44
	5–7	63	46.66
	8 or more	12	8.88
Urine pH	<5	1	0.74
	5.0–5.5	16	11.85
	5.5–6.9	50	37.03
	7.0–8.0	67	49.62
	>8	1	0.74

Body mass index (BMI); Waist-to-hip ratio (WHR); waist-to-height ratio (WHtR); Systolic Blood Pressure(SBP); Diastolic Blood Pressure(DBP).

#### Dietary assessment.

Dietary intake data were collected using multiple 24-hour dietary recalls administered over three non-consecutive days per participant, including one weekend day to capture variations in habitual dietary intake. The recalls were interviewer-administered by trained researchers using a structured interview format to ensure consistency and reduce reporting variability.

Standardized household measures, food models, and visual portion size guides were used to assist participants in accurately estimating portion sizes. When available, local food preparation methods and commonly consumed portion sizes were also considered to improve accuracy in reporting mixed dishes.

To minimize recall bias, participants were guided through a structured multiple-pass interview technique, which included repeated probing for forgotten foods, beverages, and cooking ingredients. Additionally, participants were encouraged to report intake immediately after consumption whenever possible to reduce memory-related errors. All collected dietary data were subsequently analyzed using Food Processor software (ESHA Research, Version 7).

#### Urine pH.

After participants agree to participate, urine pH test strips are sent along with an instruction manual on how to use them, and the result shown in the questionnaire is recorded. Urine pH was determined from a midstream morning urine sample using pH indicator strips (MEDICON). Urine pH was measured from a midstream morning urine sample using pH indicator strips (range 4.5–9.0; Merck, Germany). For analysis, urine pH was categorized as <5.0, 5.0–5.5, 5.5–6.9, and 7.0–8.0 [11].

To enhance the validity and reliability of self-reported measurements, all participants received standardized step-by-step written instructions and visual guidance materials developed according to validated protocols used in large epidemiological studies, including NHANES guidelines. These instructions covered standardized procedures for weight, height, waist and hip circumference measurements, and blood pressure monitoring. For blood pressure assessment, participants were instructed to use validated home sphygmomanometers and to take 3–4 repeated readings at rest, with the average value used for analysis to reduce random measurement error. Similarly, participants were guided on proper positioning and timing of anthropometric measurements to ensure consistency across respondents. For urine pH assessment, participants were provided with commercially available pH indicator strips and detailed instructions for collecting a midstream morning urine sample under standardized conditions. Although measurements were self-administered, these procedures were implemented to minimize variability and improve measurement consistency across the sample.

#### Fieldwork.

Fieldwork was conducted between 01/03/2025 and 07/07/2025across different regions of Saudi Arabia using a remote data collection approach. Participants were recruited through electronic invitations distributed via social media platforms, enabling broad geographic coverage of young women.

Upon accessing the study link, participants were presented with an information sheet outlining the study purpose and procedures, followed by an electronic informed consent form. Only those who met the eligibility criteria and provided consent were allowed to proceed.

Data collection was carried out using a structured, self-administered questionnaire. Participants were guided through each section of the survey, which included demographic characteristics, dietary intake, water consumption, anthropometric measurements, and blood pressure readings. Clear, standardized instructions were provided within the questionnaire to ensure accurate and consistent reporting.

For urine pH assessment, test strips were provided to participants along with detailed instructions on proper use. Participants were instructed to measure urine pH using a midstream morning urine sample and to record the result directly in the questionnaire.

Throughout the data collection period, responses were monitored to ensure completeness and adherence to the study criteria. Incomplete or inconsistent entries were excluded from the final dataset. Data collection continued until the required sample size (n = 135) was achieved.

### Statistical analysis

All statistical analyses were performed using IBM SPSS 26 Statistics software. Descriptive statistics were used to summarize participants’ sociodemographic, anthropometric, dietary, and cardiometabolic characteristics. Continuous variables were presented as means and standard deviations, while categorical variables were reported as frequencies and percentages.

Pearson correlation analysis was conducted to examine bivariate relationships between dietary intake variables and cardiometabolic outcomes, including systolic blood pressure, diastolic blood pressure, and urine pH. Prior to regression analysis, assumptions of linearity, normality, and absence of multicollinearity were assessed. Multiple linear regression analyses were performed to identify independent dietary predictors of systolic blood pressure, diastolic blood pressure, and urine pH. All dietary variables were entered simultaneously based on a priori theoretical and empirical evidence from previous nutritional epidemiology studies, rather than using stepwise selection procedures, to ensure hypothesis-driven modeling.

To assess potential multicollinearity among independent variables, Variance Inflation Factors (VIF) and tolerance values were calculated for all regression models. VIF values ranged between 1.3 and 3.1 across all predictors, indicating low to moderate collinearity and confirming the stability of the regression coefficients. A VIF threshold of <5 was considered acceptable. Model fit was evaluated using the coefficient of determination (R^2^), which ranged from 0.275 to 0.40 across the regression models, indicating moderate explanatory power. Statistical significance was set at p < 0.05.

### Ethical considerations

The study protocol was reviewed and approved by the Scientific Research Ethics Committee at Princess Nourah bint Abdulrahman University (Approval No.: 25-0500). All procedures were conducted in accordance with the principles of the Declaration of Helsinki for research involving human subjects. Participation in the study was entirely voluntary. Prior to data collection, all participants were provided with detailed information about the study objectives, procedures, and their rights as participants. Informed consent was obtained electronically from all participants before enrollment.

Participants were assured of the confidentiality and anonymity of their data. No personally identifiable information was collected, and all responses were stored securely and used solely for research purposes. Participants were informed that they had the right to withdraw from the study at any time without any consequences. Additionally, participants were provided with clear instructions for all self-reported measurements, including anthropometric data, blood pressure, and urine pH assessment, to ensure safe and appropriate data collection practices.

## Results

This sample included 135 Saudi female adults (19–30 years). As shown in Table 1, The descriptive analysis revealed a mixed anthropometric and cardiometabolic profile among the participants. Based on BMI classification, 33.30% of women were underweight, 25.90% had normal weight, 23.70% were overweight, and 17.00% were obese, indicating that 40.70% of the sample fell within the overweight/obese range. With respect to central adiposity, 26.60% of participants had a waist-to-hip ratio (WHR) > 0.85, while 35.50% had an elevated waist-to-height ratio (WHtR ≥ 0.50), including 5.90% at very high risk (WHtR ≥ 0.60).

Blood pressure findings showed that 59.30% of participants had diastolic blood pressure in the 80–89 mmHg range and 13.30% had DBP ≥ 90 mmHg. For systolic blood pressure, 31.1% were in the 120–129 mmHg range, while 11.8% had systolic hypertension (SBP ≥ 130 mmHg). Regarding dietary-related factors, 30.40% of participants consumed sodium at or above 2000 mg/day. Daily water intake was generally low, with 44.40% consuming only 2–5 cups/day and only 8.9% reporting intake of 8 or more cups/day. Urine pH distribution showed that nearly half of the participants (49.60%) had alkaline urine (pH 7.0–8.0), whereas 11.90% had moderately acidic urine (pH 5.0–5.5).

Demographic data reveals a population that is predominantly single (76.30%) and highly educated, with 77.80% having attained higher education. Only a very small proportion had secondary education or less. For Economic status, about 63.70% of participants earn more than 10,000 SAR monthly, classifying them as high-income individuals. The remaining participants are mostly in the upper-middle and lower-middle income brackets, with only 0.70% in the low-income category, see Table 1.

The Table 2. shows mean ± standard deviation of dietary intake. Macronutrient distribution indicated that carbohydrates contributed the largest proportion of energy intake (73.70%), followed by protein (21.40%) and fat (4.90%), demonstrating a carbohydrate-dominant dietary pattern. In addition, the majority of participants (98.50%) consumed more than 65% of their total energy from carbohydrates, exceeding the recommended acceptable macronutrient distribution range (45–65%), while only 1.50% were within the recommended range. Micronutrient intake was generally low across most vitamins and minerals, particularly omega-3 fatty acids (0.005 ± 0.007 g/kg/day) and fiber (0.18 ± 0.11 g/kg/day). Fluid intake was also suboptimal, with an average water intake of 1.64 ± 0.64 cups/kg/day.

**Table 2:** Dietary intake of macronutrients, micronutrients, fluids, and energy distribution.

Category	Nutrient	Mean ± SD
Macronutrients	Carbohydrates (g/kg/day)	3.17 ± 1.74
	Protein (g/kg/day)	0.85 ± 0.66
	Total fat (g/kg/day)	0.89 ± 0.43
	Fiber (g/kg/day)	0.18 ± 0.11
	Soluble fiber (g/kg/day)	0.02 ± 0.02
	Omega-3 fatty acids (g/kg/day)	0.005 ± 0.007
Fluids	Water intake (cups/kg/day)	1.64 ± 0.64
Vitamins	Thiamine (mg/kg/day)	0.013 ± 0.01
	Riboflavin (mg/kg/day)	0.017 ± 0.01
	Niacin (mg/kg/day)	0.18 ± 0.13
	Pyridoxine (mg/kg/day)	0.18 ± 0.13
	Folic acid (µg/kg/day)	2.67 ± 2.01
	Cobalamin (µg/kg/day)	0.024 ± 0.05
Minerals	Sodium (mg/kg/day)	26.79 ± 17.44
	Potassium (mg/kg/day)	65.74 ± 138.06
Energy distribution	Energy from carbohydrates	73.7%
	Energy from protein	21.4%
	Energy from fat	4.9%
Carbohydrate adequacy	>65% of energy intake	98.5%
	Within AMDR (45–65%)	1.5%

### Analysis of dietary predictors of systolic and diastolic blood pressure and urine pH

Multiple linear regression (Table 3) analysis identified several dietary factors associated with blood pressure and urine pH. For systolic blood pressure (SBP), higher energy intake (B = 2.53, p = 0.048), sodium intake (B = 1.02, p < 0.001), and omega-6 fatty acid intake (B = 2.08, p = 0.049) were significant positive predictors, whereas carbohydrate intake (B = –1.61, p = 0.039) and phosphorus intake (B = –0.54, p = 0.045) were inversely associated with SBP.

**Table 3:** Multiple linear regression analysis of dietary predictors of systolic and diastolic blood pressure and urine pH.

Outcome	Predictor	B	SE	95% CI	p-value	VIF
Systolic blood pressure R^2^ = 0.325	Energy Intake	2.53	1.28	0.02 to 5.05	0.048	1.7
	Carbohydrate Intake	–1.61	0.78	–3.15 to –0.08	0.039	1.3
	Pantothenic Acid	0.62	0.27	0.09 to 1.15	0.021	2.4
	Phosphorus Intake	–0.54	0.27	–1.07 to –0.01	0.045	3.1
	Sodium Intake	1.02	0.29	0.44 to 1.60	<0.001	1.9
	Omega-6 Intake	2.08	1.05	0.01 to 4.15	0.049	2.0
	Fiber Intake	–0.45	0.25	–0.96 to 0.04	0.073	1.4
	Polyunsaturated Fats	–1.99	1.09	–4.13 to 0.14	0.067	3.1
Diastolic blood pressure R^2^ = 0.40	Energy Intake	3.48	1.17	1.18 to 5.77	0.003	2.6
	Carbohydrate Intake	–2.24	0.72	–3.67 to –0.82	0.002	1.5
	Fat Intake	–1.46	0.60	–2.65 to –0.27	0.016	2.2
	Pantothenic Acid Intake	0.55	0.24	0.06 to 1.03	0.025	1.9
	Fiber Intake	–0.43	0.23	–0.89 to 0.02	0.064	2.8
	Vitamin B2 Intake	–0.30	0.16	–0.62 to 0.01	0.061	1.7
	Vitamin B6 Intake	1.04	0.57	–0.08 to 2.16	0.069	2.3
	Omega-3 Intake	–0.36	0.21	–0.77 to 0.05	0.089	2.9
	Niacin Intake	–0.97	0.55	–2.05 to 0.11	0.077	3.1
Urine pH R^2^ = 0.275	Energy Intake	–0.13	0.06	–0.27 to –0.002	0.046	1.8
	Carbohydrate Intake	0.68	0.33	0.03 to 1.33	0.040	1.9
	Omega-6 Intake	0.11	0.05	0.01 to 0.23	0.040	1.6
	Magnesium Intake	–0.41	0.14	–0.71 to –0.13	0.005	2.0
	Pantothenic Acid	–21.88	12.58	–46.54 to 2.77	0.082	1.6
	Trans Fat Intake	–10.38	5.69	–21.54 to 0.77	0.068	2.4
	Calcium Intake	–0.04	0.02	–0.09 to 0.01	0.085	2.6

For diastolic blood pressure (DBP), higher energy intake was significantly associated with increased DBP (B = 3.48, p = 0.003), while carbohydrate intake (B = –2.24, p = 0.002) and fat intake (B = –1.46, p = 0.016) were significantly associated with lower DBP. Pantothenic acid intake also showed a positive association with DBP (B = 0.55, p = 0.025).

Regarding urine pH, higher energy intake was associated with a lower (more acidic) urine pH (B = –0.13, p = 0.046), whereas carbohydrate intake (B = 0.68, p = 0.040) and omega-6 intake (B = 0.11, p = 0.040) were associated with higher (more alkaline) urine pH. Magnesium intake demonstrated a strong inverse association with urine pH (B = –0.41, p = 0.005). Several variables, including fiber intake, polyunsaturated fat, vitamin B2, vitamin B6, omega-3 fatty acids, niacin, trans fat, and calcium, showed non-significant trends toward associations with the studied outcomes.

Pearson correlation analysis (Table 4) demonstrated that caffeine intake was significantly and positively associated with energy intake (r = 0.31, p < 0.05), indicating that individuals with higher caffeine consumption tended to have higher overall caloric intake. However, caffeine intake was not significantly associated with urine pH, blood pressure (SBP and DBP), body mass index (BMI), or anthropometric measures (HC and WC).

**Table 4:** Pearson correlation matrix of caffeine intake and selected cardiometabolic and anthropometric parameters.

Variables	Caffeine	Urine pH	DBP	SBP	HC	WC	Energy intake	BMI
Caffeine intake	1	0.03	–0.07	–0.02	–0.10	–0.13	0.31*	0.01
Urine pH		1	–0.16	–0.01	–0.09	–0.06	0.03	0.04
DBP			1	0.54*	0.13	0.08	0.24*	0.01
SBP				1	0.08	0.002	0.13	0.09
HC					1	0.87*	0.14	0.36*
WC						1	0.14	0.38*
Energy intake							1	0.25*
BMI								1

Body mass index (BMI);Waist circumference (WC); hip circumference (HC); Waist-to-hip ratio (WHR); waist-to-height ratio (WHtR); Systolic Blood Pressure(SBP); Diastolic Blood Pressure(DBP). and ^b^ indicate statistically significant differences (P < 0.05).

A strong positive correlation was observed between systolic and diastolic blood pressure (r = 0.54, p < 0.05). Additionally, hip circumference was strongly correlated with waist circumference (r = 0.87, p < 0.05), and both measures were positively associated with BMI (r = 0.36 and r = 0.38, respectively; p < 0.05). Energy intake showed a significant positive correlation with both diastolic blood pressure (r = 0.24, p < 0.05) and BMI (r = 0.25, p < 0.05). No significant correlations were observed between urine pH and the examined variables.

### Pearson correlation between urine pH and nutrient intakes

As shown in Table 5. Pearson correlation analysis showed generally weak associations between urine pH and dietary nutrient intakes. Among the examined nutrients, soluble fiber intake demonstrated a statistically significant positive correlation with urine pH (r = 0.18, p = 0.031), indicating that higher soluble fiber consumption was associated with a more alkaline urine pH. Similarly, thiamin (vitamin B1) intake was positively correlated with urine pH (r = 0.19, p = 0.023).

**Table 5:** Pearson correlations between urine pH and dietary nutrient intakes among young saudi women (n = 135).

Nutrient Intake	r	p-value
Protein	0.06	0.492
Carbohydrate	0.05	0.542
Fiber	0.14	0.091
Soluble Fiber	0.18	0.031
Fat	0.04	0.594
Saturated Fat	0.08	0.359
Monounsaturated Fat	0.09	0.288
Polyunsaturated Fat	0.03	0.686
Cholesterol	0.03	0.677
Trans Fat	–0.01	0.870
Thiamin (Vitamin B1)	0.19	0.023
Riboflavin (Vitamin B2)	0.07	0.382
Niacin (Vitamin B3)	0.15	0.070
Pyridoxine (Vitamin B6)	0.15	0.070
Cobalamin (Vitamin B12)	–0.03	0.650
Folic Acid	0.07	0.405
Pantothenic Acid	0.02	0.816
Calcium	–0.04	0.634
Iron	0.06	0.461
Magnesium	0.03	0.654
Manganese	0.05	0.561
Phosphorus	0.03	0.747
Potassium	0.06	0.430
Sodium	0.139	0.107
Zinc	0.124	0.152
Omega-3	0.053	0.543
Omega-6	0.024	0.782
Caffeine	0.033	0.705
Selenium	0.067	0.443

All other nutrients, including protein, total carbohydrate, fat and its subtypes, cholesterol, vitamins, and minerals, showed no statistically significant associations with urine pH (p > 0.05). Although some variables, such as total fiber, niacin, and pyridoxine, exhibited positive trends, these associations did not reach statistical significance.

## Discussion

Cardiometabolic risk factors have been extensively studied due to their increasing global burden and their association with adverse cardiovascular outcomes [4]. In this context, the present study examined the relationships between dietary intake patterns, water consumption, urine pH, and selected cardiometabolic risk indicators among young Saudi women. The results indicate potential associations between dietary composition and key health indicators, including blood pressure and anthropometric measures. Notably, this study contributes to the limited evidence in Saudi Arabia by integrating dietary assessment, hydration status, urine pH, and multiple cardiometabolic risk indicators within a single analytical framework in a young female population.

The findings indicate that a substantial proportion of participants were either overweight or obese (approximately 41%), with central obesity observed in 15.6% of the sample. These observations are consistent with previous reports highlighting the high prevalence of excess body weight among adults, which has been linked to shifts toward energy-dense dietary patterns and reduced physical activity levels [12,13]. Additionally, nearly half of the participants exhibited elevated or hypertensive blood pressure levels, which is concerning in a relatively young population, as early elevations in blood pressure have been associated with increased long-term cardiovascular risk [14].

Patterns of urine pH observed in this study may suggest potential variations in dietary acid–base balance and hydration status; however, these findings should be interpreted cautiously, as urine pH is influenced by multiple dietary and physiological factors. Taken together, these results highlight important associations between dietary patterns and cardiometabolic indicators in young adulthood. Nevertheless, due to the cross-sectional design of the study, causal inferences cannot be made. These findings support the need for future preventive strategies focusing on improving dietary quality and lifestyle behaviors to reduce the burden of noncommunicable diseases in this population.

The dietary analysis revealed that the majority of participants derived more than 65% of their total energy from carbohydrates, exceeding the acceptable macronutrient distribution range. Such excessive reliance on carbohydrates, particularly in the context of low fat and protein intake, may contribute to dyslipidaemia, weight gain, and other health issues [15]. More than 40% of participants consumed sodium above the recommended level, indicating a potential risk factor for elevated blood pressure and cardiovascular disease [5]. This finding aligns with the observation that nearly half of the participants presented with elevated or hypertensive blood pressure values as mentioned above. Additionally, inadequate fluid intake was observed, with 44.4% of participants consuming fewer than four cups of water per day, far below recommended hydration guidelines [7]. Low water intake has been associated with concentrated urine, reduced urine pH, and a higher risk of urinary tract complications and kidney stone formation [16]. Adequate hydration supports kidney function and fluid balance; higher water intake is associated with slower progression of chronic kidney disease [17]. Therefore, the combination of high carbohydrate consumption, excessive sodium intake, and suboptimal hydration may synergistically contribute to overweight prevalence, hypertension, and the abnormal urine pH values observed in this study, reflecting early metabolic and renal health risks among Young Saudi women.

The correlation analyses reinforce and elaborate upon the descriptive findings, demonstrating that higher total energy and sodium intake are robustly associated with elevated blood pressure, while dietary fiber, certain vitamins, and heart-healthy fats (polyunsaturated and omega-3 fatty acids) appeared to exert protective effects on blood pressure regulation. These observations are consistent with previous reviews that reported significant associations between sodium, fiber, and fat intake and the risk of blood pressure-related disorders [5]. Regarding urine pH, higher energy intake was modestly associated with lower urine pH (B = –0.136, p = 0.046), suggesting a potential relationship between increased caloric consumption and a more acidic urinary environment. This observation may reflect broader dietary acid–base balance rather than a direct effect of energy intake alone, as previously suggested in nutritional acid-load literature [18,19]. In addition, magnesium intake showed an inverse association with urine pH (B = –0.416, p = 0.005); however, this finding should be interpreted cautiously given the complex interactions between dietary minerals, overall dietary patterns, and renal regulation of acid–base homeostasis.

Conversely, fiber intake and certain B-vitamins showed trends toward higher urine pH, which may reflect overall healthier dietary patterns typically associated with increased consumption of plant-based foods, which have been linked to more alkaline urine profiles in previous studies [19]. Nevertheless, these associations should not be interpreted as causal effects of individual nutrients, but rather as components of broader dietary patterns that collectively influence systemic acid–base balance.

Overall, these findings align with the descriptive results and suggest that dietary composition, particularly the balance between energy-dense and nutrient-rich foods, plays a more important role in blood pressure regulation and urine pH than isolated nutrient effects. However, given the cross-sectional design and potential residual confounding among dietary variables, these results should be interpreted as exploratory and hypothesis-generating.

### Limitations

A limitation of this study is the use of a convenience sample recruited through electronic invitations, which may introduce selection bias and limit generalizability to the broader population of young Saudi women. Individuals without access to digital platforms or those less engaged with online systems may have been underrepresented. Therefore, the findings should be interpreted as exploratory and hypothesis-generating rather than population-representative estimates.

The data on dietary intake, water consumption, anthropometric measures, and blood pressure were self-reported, which may introduce recall bias and measurement inaccuracies. Although standardized instructions were provided, variability in home-based measurements cannot be excluded. Fourth, dietary assessment was based on 24-hour recalls, which may not fully reflect habitual intake despite attempts to include multiple days.

Additionally, urine pH was measured using test strips under non-clinical conditions, which may be less precise compared to laboratory-based methods. Finally, potential confounding factors such as physical activity, stress levels, and genetic predisposition were not fully controlled. Future studies with larger, randomly selected samples and longitudinal designs are recommended to validate and expand these findings. Measurement error may still exist due to the self-administered nature of data collection despite the use of standardized instructions and repeated measurements.

## Conclusion

Dietary intake patterns and hydration status were significantly associated with blood pressure and urine pH among young Saudi women. In multiple regression analyses, higher energy and sodium intake were positively associated with systolic and diastolic blood pressure, while carbohydrate intake showed inverse associations with blood pressure outcomes. Additionally, dietary fiber, selected vitamins, and unsaturated fatty acids demonstrated variable associations with blood pressure regulation.

Regarding urine pH, higher energy intake was associated with lower urine pH, whereas carbohydrate intake and omega-6 fatty acids showed positive associations. Magnesium intake demonstrated a significant inverse association with urine pH. Correlation analyses further indicated weak to moderate associations between dietary intake, anthropometric measures, and blood pressure, while associations with urine pH were generally weaker.

Overall, the findings suggest moderate associations between dietary composition and cardiometabolic risk indicators in this population. However, given the cross-sectional design, reliance on self-reported dietary and measurement data, and potential residual confounding, these findings should be interpreted strictly as exploratory associations and not as evidence of causality or disease development.

The observed dietary patterns characterized by high carbohydrate and sodium intake with low fiber and unsaturated fat consumption highlight potential areas for nutritional improvement in early adulthood. These results may help generate hypotheses for future longitudinal studies aimed at clarifying temporal relationships between dietary factors, blood pressure regulation, and urinary acid–base balance.
